# Long-term taxonomic and functional stability of the gut microbiome from human fecal samples

**DOI:** 10.1038/s41598-022-27033-w

**Published:** 2023-01-03

**Authors:** Jae Hyun Kim, Ji-Young Jeon, Yong-Jin Im, Na Ha, Jeon-Kyung Kim, Seol Ju Moon, Min-Gul Kim

**Affiliations:** 1 School of Pharmacy and Institute of New Drug Development, Jeonbuk National University, Jeonju, Republic of Korea; 2 Center for Clinical Pharmacology and Biomedical Research Institute, Jeonbuk National University Hospital, 20, Geonji-ro, Deokjin-gu, Jeonju-si, Jeollabuk-do 54907 Republic of Korea; 3 Department of Pharmacology, Medical School, Jeonbuk National University, Jeonju, Republic of Korea; 4 Research Institute of Clinical Medicine of Jeonbuk National University, Jeonju, Republic of Korea

**Keywords:** Microbial communities, Clinical trial design

## Abstract

Appropriate storage of fecal samples is a critical step for unbiased analysis in human microbiome studies. The purpose of this study was to evaluate the stability of the fecal microbial community for up to 18 months. Ten healthy volunteers provided fecal samples at the Jeonbuk National University Hospital. Stool samples were stored under the following six conditions: four different storage temperatures (− 70 °C, − 20 °C, 4 °C, and room temperature [20–25 °C]) and two different collection tubes (OMNIgene-Gut and DNA/RNA shield-fecal collection tubes). The gut microbiome was analyzed with 16S rRNA sequencing. We compared the taxonomic composition, alpha diversity, beta diversity and inferred pathway abundance between the baseline and 18 months after storage. Samples collected in the DNA/RNA Shield-fecal collection tubes showed the best performance in preservation of the taxonomic composition at 18 months. Pairwise differences in alpha diversity metrics showed the least deviation from zero. The PERMANOVA test showed non-significant change of beta diversity metrics (Unweighted Unifrac: q-value 0.268; Weighted Unifrac: q-value 0.848). The functional stability was significantly well preserved in the DNA/RNA Shield-fecal collection tubes (adjusted p value < 0.05). Our results demonstrate the use of the DNA/RNA Shield-fecal collection tube as an alternative storage method for fecal samples to preserve the taxonomic and functional stability of the microbiome over a long term.

## Introduction

The human gut microbiome is a group of microorganisms consisting of bacteria, archaea, and eukarya, and the number of microorganisms in the human gastrointestinal tract is estimated to be about ~ 10^14^ for a male of 70 kg^1^. The gut microbiome plays a major role in maintaining host homeostasis by modulating the immune system and metabolism^2,3^. Studies on the function of the microbiome will be critical to understanding the role of the microbiome in human homeostasis and disease pathogenesis^2^.

Specimens obtained from human feces are the most commonly used as a proxy for the human gut microbiome research^4^. Fecal sampling methods have a few advantages in that they are noninvasive, inexpensive, and more convenient than other invasive methods, such as biopsy^4^. However, the detected composition of the fecal microbial community can be affected by the experimental design and procedures, including the sampling method, storage condition and reference database used^5,6^. As differences in stool collection methods contribute to interstudy variability, gut microbiome studies should utilize valid, reproducible, and standardized methods to preserve the microbiome composition and to enhance data comparability.

Studies have shown that the gut microbiome changes significantly during storage under ambient temperature^7,8^. As a result, immediate freezing and storage at − 80 to − 70 °C have been widely accepted as best practices for sequence-based analyses^9^. However, immediate freezing using a deep freezer is usually impractical in the clinical setting^7,10^. Alternative methods, such as storage at room temperature (20–25 °C), refrigeration (4 °C), freezing (− 20 °C) or the usage of collection tubes with preservatives, are considered in practice. Different studies have investigated the temporal variability of a microbial community under different storage conditions to confirm whether any of the storage conditions show comparable stability^7,10–18^. However, these studies were limited in terms of sample size, storage duration, and tested microbial endpoints. Some studies have tested different storage conditions with fewer than five subjects^7,11,12,14^. Other studies with even more subjects often had a short-term follow-up of less than a year^10,13,15,16^. The tested microbial endpoints were variable but mostly confined to taxonomic stability, such as a change in taxonomic composition and diversity metrics during storage.

Therefore, the purpose of our research was to investigate the taxonomic and functional stability of the microbiome from human fecal samples over a long term. We evaluated the taxonomic composition, diversity, and inferred pathway abundance at 18 months after storage depending on the various storage temperatures and collection kits.

## Results

A total of ten volunteers participated in the clinical trial. The median age of the volunteers was 24 years (interquartile range [IQR] 23–28.75 years), and all of the volunteers were male. The median body mass index of the volunteers was 24.5 kg/m^2^ (IQR 21.8–27.7 kg/m^2^). Sixty samples from homogenized stool samples were obtained from ten volunteers. Samples obtained from homogenized stools were stored until 18 months. The quantity of stool contents obtained from one volunteer each belonging to the DNA/RNA Shield group and the OMNIgene-Gut group was not sufficient, thereby leading to their exclusion from further analysis. Therefore, a total of 58 samples were used in further analysis.

**Figure 1:**
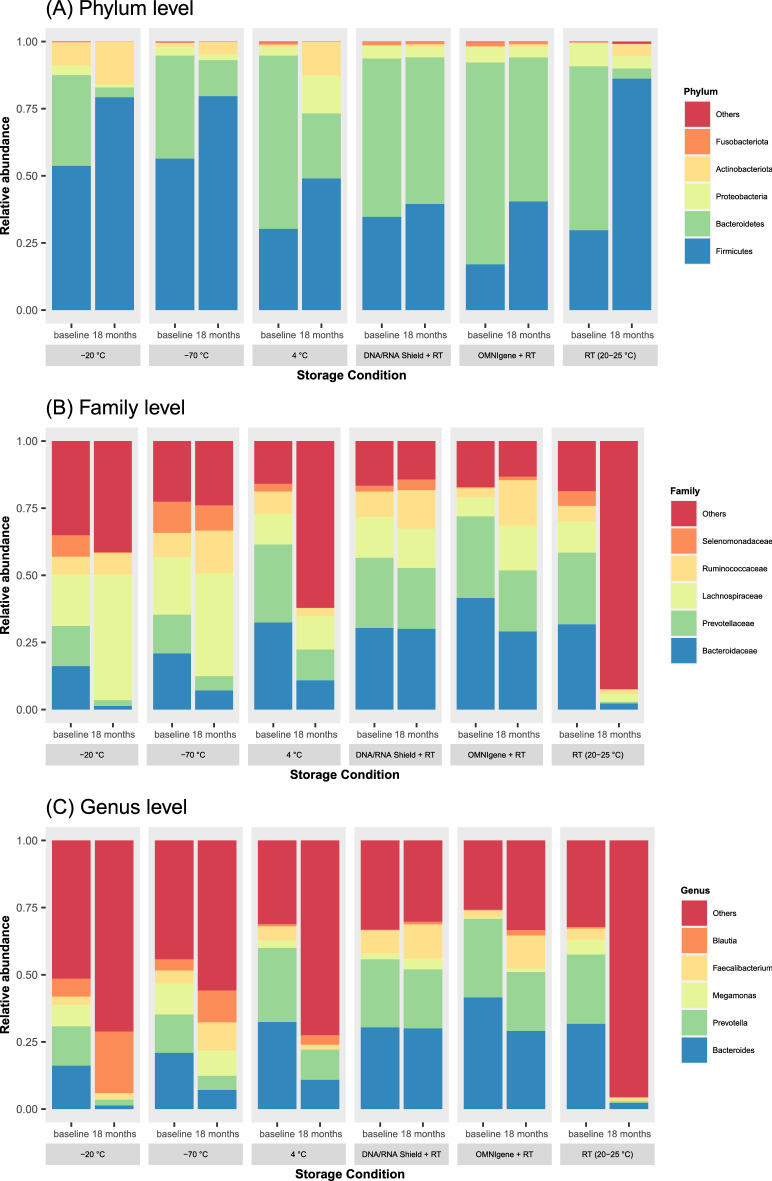
Comparison of the taxonomic composition at the (**A**) phylum, (**B**) family and (**C**) genus levels according to the tested storage conditions. *DNA/RNA Shield* DNA/RNA shield-fecal collection tube, *OMNIgene* OMNIgene-Gut, *RT* room temperature (20–25 °C).

The quality of forward and reverse sequence reads is presented in Supplementary Fig. S1. The median quality score of reads was above 30 until the 188th and 221st positions of the forward and reverse sequence reads, respectively. The taxonomic composition at the phylum, family, and genus levels according to the storage conditions is presented in Fig. 1. The top five most abundant phyla, families and genera were selected, and the remaining taxa were aggregated and described as the others in Fig. 1 to facilitate visual comparison between the tested storage conditions. The taxonomic composition was most preserved in the DNA/RNA Shield group at phylum, family, and genus levels. During sample storage, the relative abundance of the phylum *Bacteroidetes* decreased at 18 months in all the tested storage conditions, while the relative abundance of the phylum *Firmicutes* increased. The decrease in the relative abundance of the phylum *Bacteroidetes* was pronounced, especially in the room temperature group.

Supplementary Figure S2 shows the taxonomic composition of samples from homogenized stools and non-homogenized stools at baseline. The relative abundance data from 10 healthy volunteers were averaged in Supplementary Fig. S2. The effect of homogenization on the taxonomic composition was negligible (Supplementary Fig. S2B vs. C). However, samples from homogenized stools that were stored in the DNA/RNA Shield group at room temperature showed different taxonomic compositions when compared to samples stored under − 70 °C.

**Figure 2:**
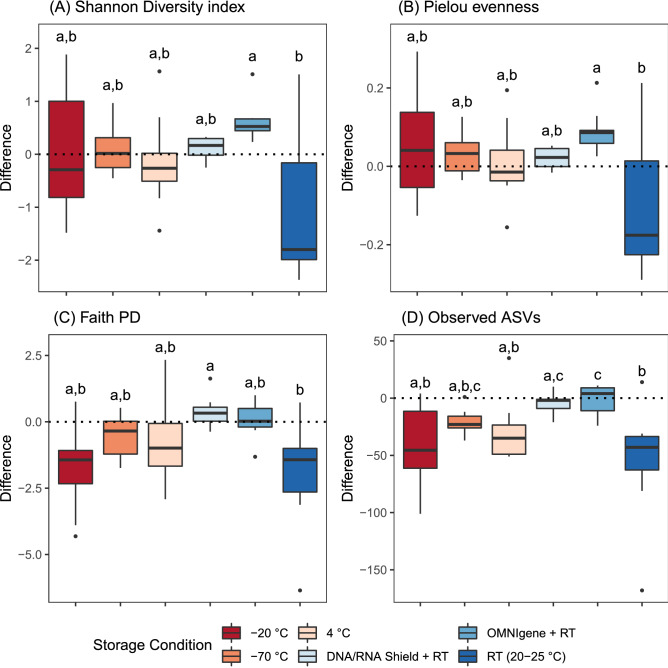
Pairwise differences in alpha diversity metrics between the baseline and 18 months. *DNA/RNA Shield* DNA/RNA shield-fecal collection tube, *OMNIgene* OMNIgene-Gut, *RT* room temperature (20–25 °C). Groups that do not share any letter denote statistical difference by the Dunn’s test at the 5% level of significance. The legend is shared across plots.

Before the calculation of alpha and beta diversity metrics, sequences were rarefied at 31,895 sequences per sample without replacement. The level of sampling depth was determined as the minimum number of feature counts after the denoising step. The pairwise differences in alpha diversity metrics according to different storage conditions are presented in Fig. 2. The median pairwise differences in Shannon Diversity index, Pielou evenness, and observed amplicon sequence variants (ASVs) were closest to 0 in the DNA/RNA Shield group among samples stored in the room temperature (Fig. 2A,B,D). Regarding Faith phylogenetic diversity (PD), samples from the OMNIgene-Gut group showed the most stable results (Fig. 2C). In all of the tested alpha diversity metrics, significant difference between groups was observed according to the Kruskal–Wallis test (p < 0.05). In the post hoc analysis, samples stored in the room temperature showed significant difference when compared to samples stored in the collection tubes (Fig. 2A, OMNIgene group vs. RT group, p < 0.05; Fig. 2B, OMNIgene group vs. RT group, p < 0.05; Fig. 2C, DNA/RNA Shield group vs. RT group, p < 0.05; Fig. 2D, OMNIgene group or DNA/RNA Shield group vs. RT group, p < 0.05). There was no significant difference in any of the tested alpha diversity metrics between the DNA/RNA Shield group and the OMNIgene group according to the results of the Dunn’s test.

**Figure 3:**
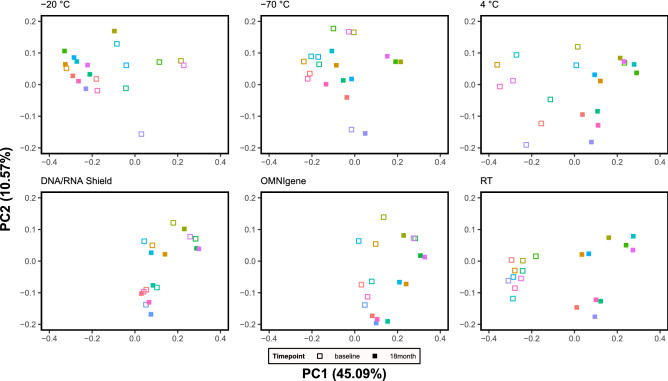
Principal coordinates analysis (PCoA) plot. Colors denote ten different subjects. *DNA/RNA Shield* DNA/RNA shield-fecal collection tube, *OMNIgene* OMNIgene-Gut, *RT* room temperature (20–25 °C).

Figure 3 shows the principal coordinates analysis plot based on the weighted UniFrac distance. The distance between samples from the two different timepoints was shortest in the samples from the DNA/RNA Shield. There was a greater dispersion in the low-dimensional space of principal coordinate analysis plots when samples were stored in conditions other than a fecal collection tube with DNA/RNA Shield. Supplementary Table S1 shows the results of the PERMANOVA test that compared baseline data with that of the 18 months for each of the storage conditions. The samples stored in the DNA/RNA Shield fecal collection tube showed a non-significant change in terms of beta diversity metrics between baseline and 18 months (Unweighted Unifrac: q-value 0.268; Weighted Unifrac: q-value 0.848).

The pairwise distance of Bray–Curtis dissimilarity of the predicted abundances of MetaCyc pathways is presented in Supplementary Fig. S3. The functional stability of the gut microbiome in the samples, which was evaluated with the change in the pathway abundance, was best preserved in the samples from the DNA/RNA Shield group (FDR adjusted p value < 0.05 for all comparisons). Four MetaCyc pathways were selected, and the relative abundances of individual pathways were compared across the tested storage conditions (Supplementary Fig. S4).

## Discussion

This research included an analysis of the long-term microbial stability of samples from human feces under six different storage conditions. The samples within the DNA/RNA Shield group were the most stable in terms of taxonomic composition, alpha diversity, beta diversity, and inferred pathway abundance. Storing the samples at room temperature without any preservatives resulted in a wide variation. The taxonomic and functional stability of the gut microbiome remained relatively unchanged in the DNA/RNA Shield group.

The addition of preservatives such as DNA/RNA Shield is known to preserve microbial diversity by inactivating bacterial growth or decay within samples^19^. Chelating agent-based solutions are used to stabilize the nucleic acid contained in feces at ambient temperatures. However, to our knowledge, it was not known how long the samples can be stored while preserving the taxonomic and functional stability even with the addition of DNA/RNA Shield. The studies to date have focused on the taxonomic stability of samples when stored for up to a few weeks or months^10,13,15,16^. In addition to previous reports regarding short-term stability, our study results provide new evidence that homogenized human stool samples could be stored at room temperature for up to 18 months when stored in a fecal collection tube with DNA/RNA Shield. A collection tube simplifies the collection process such that an individual can collect samples at home and does not require immediate storage at − 70 °C and cold chain transportation. In both clinics and decentralized research sites, a fecal collection tube will be easy to use since it does not require a cold environment and can be stored at room temperature.

The establishment of a sample storage protocol that ensures the preservation of microbial diversity for a longer period is of importance in terms of clinical trial design. Clinical trials usually recruit patients over a long period of time, and it may take more than 1 year until the completion of the trial^20^. Therefore, an evidence-based sample storage protocol is required for robust analysis of the primary or secondary endpoint of trials. For clinical trials of microbiome-based therapeutics, endpoints such as the overall compositional differences, change in relative abundances of target species, the overall change in taxonomic composition, and within- or between-group microbial diversity have been used^21,22^. More reliable analysis results would be obtained with an established sample storage methodology.

This study has additional strengths in its robust research protocol. We registered the research protocol to the Clinical Research Information Service (CRIS), an online registration platform for clinical trials, before the first enrollment of study volunteers. The research was conducted as planned in accordance with the predefined protocol, with timely amendments as needed. Our protocol was meant to minimize the interindividual variability due to variables other than the tested storage conditions. Methods including specimen collection, specimen selection, and sample handling are known factors of variation in microbiome studies, and thus, we standardized the protocol^5^. We devised and distributed the specific stool collection kits for study volunteers to easily collect the stools and to minimize the possible protocol violations at the collection step. The following measures have been taken to minimize external contamination during stool sample collection. Study volunteers were only allowed to defecate in the research bathroom in the Clinical Trial Center at Jeonbuk National University Hospital. During the study period, the toilet was disinfected prior to and after defecation and was not used for any other purpose. Laboratory staff were equipped with knowledge about human stool collection and subsequent processing.

This study is not without limitations. First, this study included analysis of samples at only two timepoints: (1) baseline and (2) 18 months after storage. The study did not include results from periods between those two timepoints. Although the microbial diversity of samples from the DNA/RNA Shield group is highly likely to be preserved even at the midpoint, questions remain about the longitudinal change in other samples. However, sample stability during the early period was not the main purpose of our study. Second, the tested storage conditions did not include more diverse alternatives. For example, samples could be stored in a deep freezer (i.e., − 70 °C) with the use of a DNA/RNA Shield fecal collection tube. However, this method was not included in our study.

This research is the first study to investigate the taxonomic and functional stability of the microbiome in samples from homogenized human stool for up to 18 months. For a clinical trial that explores microbial endpoints, human stool samples could be stored at room temperature for up to 18 months with the use of a DNA/RNA Shield fecal collection tube. Taxonomic composition and microbial diversity were well preserved under such storage conditions.

## Methods

### Clinical trial

This study was approved by the Jeonbuk National University Hospital Institutional Review Board. The clinical trial was conducted in accordance with the Declaration of Helsinki. The clinical trial protocol for this study was registered in the Clinical Research Information Service (CRIS registration ID: KCT0005101). Adult volunteers aged over 19 years who signed written informed consent were enrolled. Ten volunteers provided stool samples at the Center for Clinical Pharmacology, Jeonbuk National University Hospital.

### Fecal sample collection and processing

Stool samples were obtained immediately after their bowel movement. Before homogenization, samples were obtained from 1, 3, and 6 random regions of non-homogenized stool. And those samples from non-homogenized stool were stored under − 70 °C until the 16s rRNA sequencing. Samples from non-homogenized stools were used only to compare the effect of homogenization at baseline and were not stored for 18 months. Stools samples were homogenized immediately after obtaining samples from non-homogenized stools. For fecal sample homogenization, an amount of sterile saline solution (4 °C) that was directly proportional to the stool’s weight was added to the remaining stools, and the specimen was placed in a mixer for 10 min. The homogenized fecal samples were stored at different temperatures (− 70 °C, − 20 °C, 4 °C, and room temperature [20–25 °C]) and sequenced to provide baseline status. Additionally, homogenized fecal samples were collected using two different collection kits, the OMNIgene-Gut and DNA/RNA shield-fecal collection tubes, at room temperature. In summary, the analysis included fecal samples that were stored under the following six different storage conditions: (1) − 70 °C; (2) − 20 °C; (3) 4 °C; (4) room temperature (20–25 °C); (5) DNA/RNA Shield fecal collection tube; and (6) OMNIgene-Gut collection tube.

### DNA extraction and PCR amplification

After thawing the stool sample, bead beating to achieve lysis was performed using a FastPrep-24 (MP biomedical, USA) prior to DNA extraction. DNA was extracted using a Chemagic DNA Stool Kit (PerkinElmer, USA) and Chemagic 360 (PerkinElmer, USA) according to the manufacturer’s instructions. Prepared DNA samples were used for 16S library construction using NEXTflex 16S V4 (forward = 5′-TATGGTAATTGTGTGCCAGCMGCCGCGGTAA-3′; reverse = 5′-AGTCAGTCAGCCGGACTACHVGGGTWTCTAAT-3′) Amplicon-Seq (Bioo Scientific, USA). Paired-end sequencing was performed with a MiSeq Reagent Kit v2 Nano using a MiSeq instrument according to the manufacturer’s instructions (Illumina, USA).

### Analysis of 16S rRNA sequences

Paired-end sequences were imported in FASTQ format, and the primers were trimmed using cutadapt^23^. The quality of sequencing reads was evaluated to determine the truncation length. Both forward and reverse sequences were truncated at the point where the median quality score was less than 30 for the first time. The DADA2 method was used for denoising the sequences^24^. The appropriateness of the denoising process was evaluated by reviewing the summary results, such as the percentage of merged sequences. The taxonomy was assigned to each of the ASVs using the feature classifier supported by QIIME 2^25^. The preformatted SILVA (Release 138) reference sequences and corresponding taxonomy were used for the training of the naïve-Bayes classifier^26,27^. Input reads were classified to the species level while monitoring the classification confidence.

Sequences were rarefied before the calculation of diversity metrics. Alpha and beta diversity metrics were calculated to evaluate the within-sample and between-sample diversity. The calculated alpha diversity metrics included the Shannon Diversity index, Pielou evenness, Faith PD, and observed ASVs to account for evenness, richness, and phylogeny. The Kruskal–Wallis test was used to identify any significant difference in alpha diversity metrics among the multiple storage conditions. The Dunn’s test was used for post hoc comparison^28^. The evaluated beta diversity metrics included unweighted UniFrac distance and weighted UniFrac distance. A principal coordinates analysis plot was generated with the weighted UniFrac distance to aid the visual inspection of the sample clusters. The PERMANOVA test was used to detect differences in beta diversity metrics between multiple storage conditions.

PICRUSt2 was used to evaluate the functional stability of the microbiome^29^. The MetaCyc pathways were predicted from the representative sequences and their relative abundances^30^. Bray–Curtis dissimilarity was calculated after rarefying with the minimum number of inferred pathway abundances among samples. The overall pairwise distance of Bray–Curtis dissimilarity was compared across the sample storage methods, and the statistical significance was tested with the Kruskal–Wallis test.

### Software

The analyses of 16S rRNA sequences, including the taxonomic classification and calculation of diversity metrics, were performed using Python v3.8.10 and QIIME 2 v2021.8.0^31^. QIIME 2 and PICRUSt2 were installed within a virtual environment according to the installation documentation. R v4.0.0 was used to draw the figures with the obtained results. Codes for the analysis of 16S rRNA sequences and the generation of figures are available in the following GitHub repository: https://github.com/kimkimjh/CUH_2019_SMT.

### Ethics approval

This study was approved by the Jeonbuk National University Hospital institutional review board.

## Supplementary Information

Supplementary Information.

## Data Availability

The 16S rRNA sequences generated and/or analyzed during the study are available from the following NCBI BioProject Accession Number: PRJNA814893.

## Code availability

The analysis codes are available from the following GitHub repository: https://github.com/kimkimjh/CUH_2019_SMT.
