# Supplementary material for: Long-term taxonomic and functional stability of the gut microbiome from human fecal samples
Source: Sci Rep. 2023 Jan 3;13:114. doi: 10.1038/s41598-022-27033-w (PMC9810722; doi:10.1038/s41598-022-27033-w)
Supplement: Supplementary file 1 — Supplementary Information. [file 41598_2022_27033_MOESM1_ESM.docx]

**Supplementary Materials**

**Supplementary Figure S1.** Quality scores (Phred score) of (A) forward and (B) reverse reads.

**Supplementary Figure S2.** Comparison of the taxonomic composition between samples from (A-B) homogenized stools and (C) non-homogenized stools at baseline. Stool samples were obtained from 10 healthy volunteers.

**Supplementary Figure S3.** Pairwise distances of Bray–Curtis dissimilarity based on the predicted MetaCyc pathways between the baseline and 18 months. DNA/RNA Shield, DNA/RNA shield fecal collection tube; OMNIgene, OMNIgene-Gut; RT, room temperature.

**Supplementary Figure S4.** Relative abundances of the inferred MetaCyc pathways at the baseline and 18 months after storage. (A) Pentose phosphate pathway (nonoxidative branch) I, (B) gondoate biosynthesis (anaerobic), (C) adenosine ribonucleotides de novo biosynthesis, and (D) UMP biosynthesis I.

**Supplementary Table S1.** Pairwise comparison of beta diversity from baseline and 18 months samples by PERMANOVA


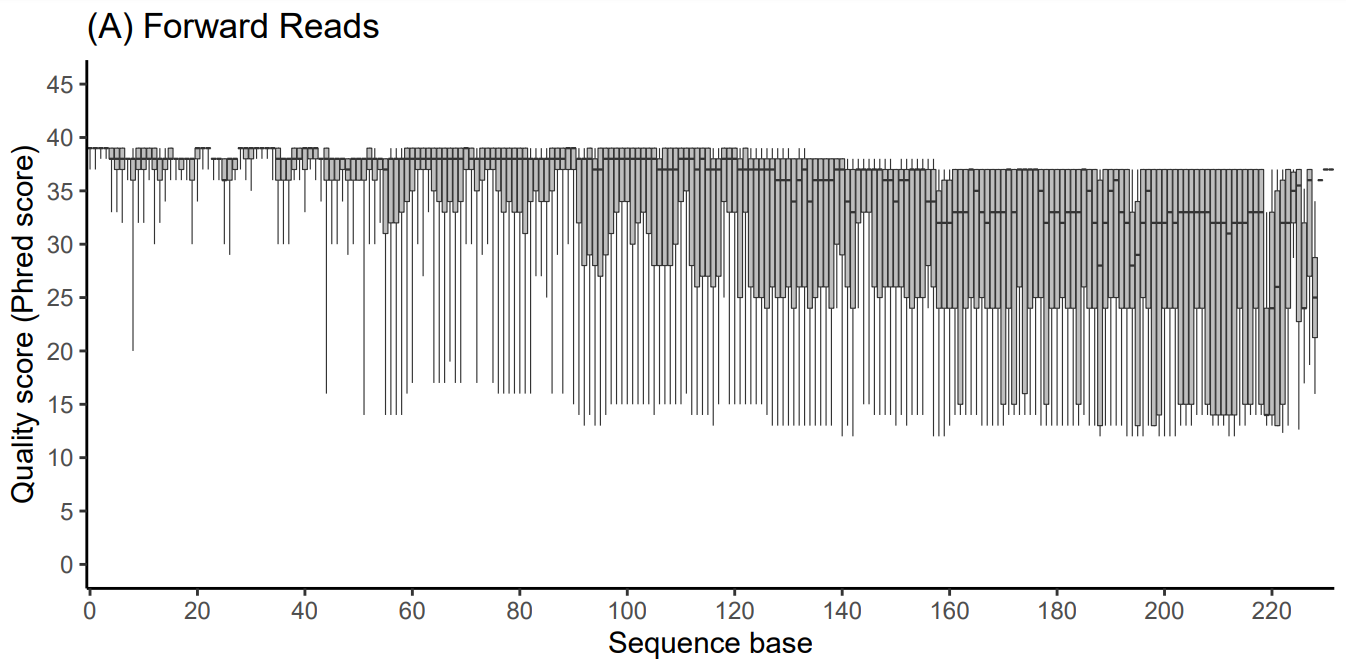


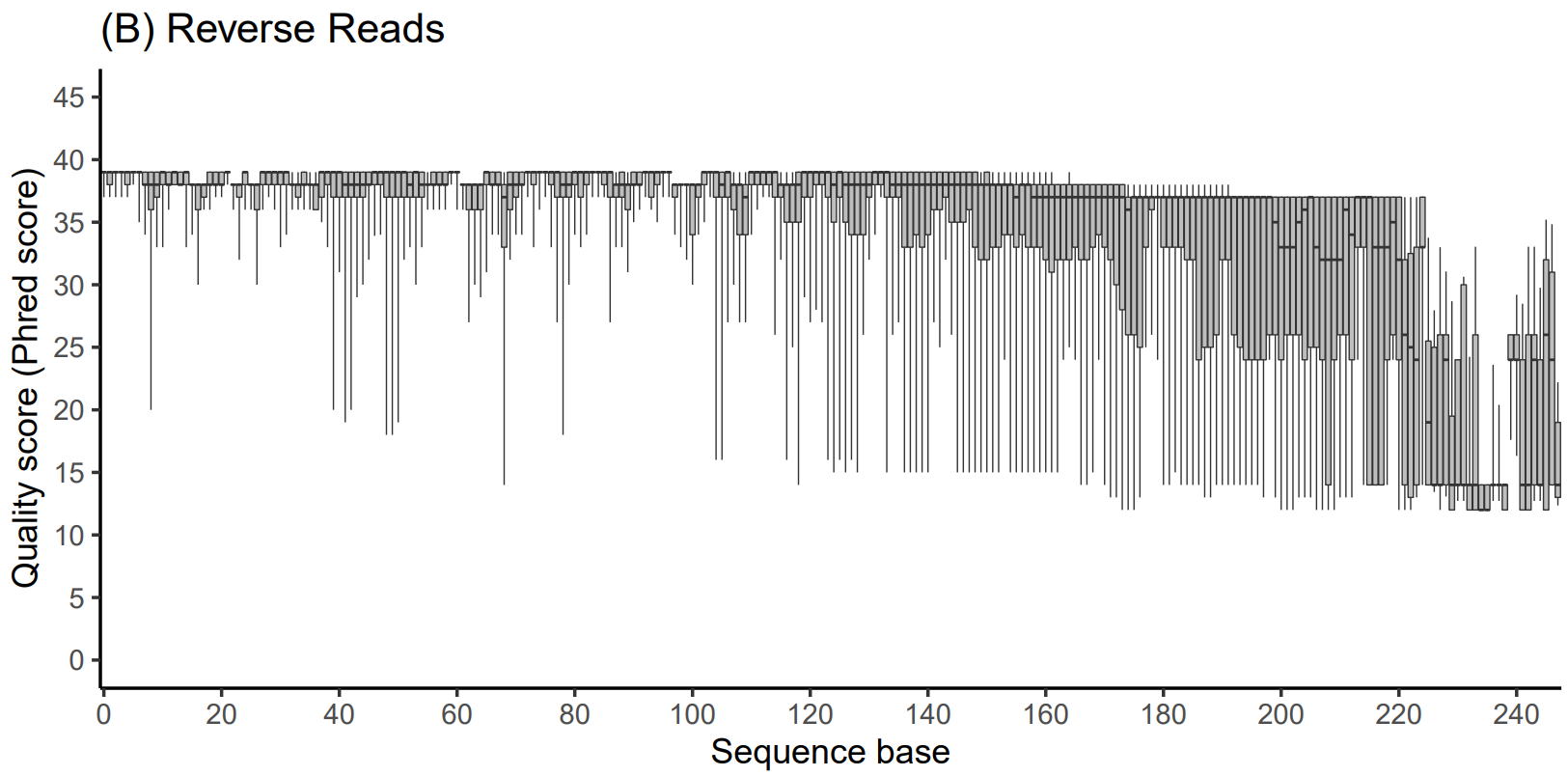


**Supplementary Figure S1.** Quality scores (Phred score) of (A) forward and (B) reverse reads.


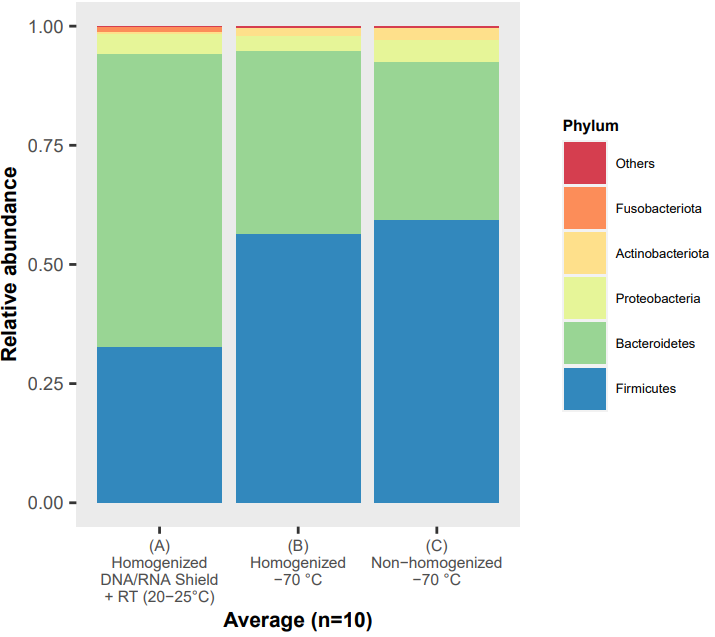


**Supplementary Figure S2.** Comparison of the taxonomic composition between samples from (A-B) homogenized stools and (C) non-homogenized stools obtained at baseline. Stool samples were obtained from 10 healthy volunteers. (A) Samples were stored in the DNA/RNA shield fecal collection tube at RT until 16s rRNA sequencing. (B-C) Samples were stored under -70 ℃ until 16S rRNA sequencing.


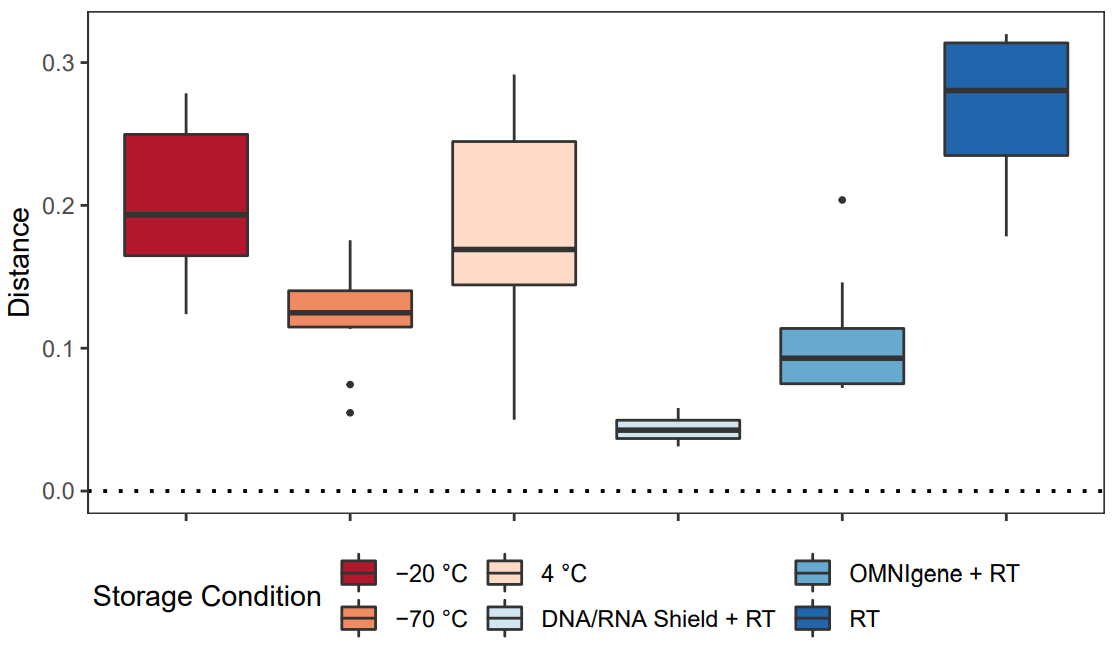


**Supplementary Figure S3.** Pairwise distances of Bray–Curtis dissimilarity based on the predicted MetaCyc pathways between the baseline and 18 months. DNA/RNA Shield, DNA/RNA shield fecal collection tube; OMNIgene, OMNIgene-Gut; RT, room temperature.


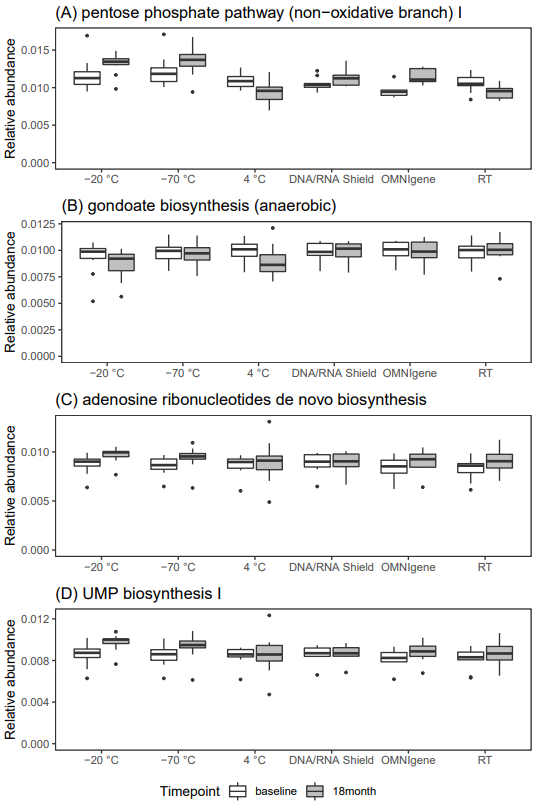


**Supplementary Figure S4.** Relative abundances of the inferred MetaCyc pathways at the baseline and 18 months after storage. (A) Pentose phosphate pathway (nonoxidative branch) I, (B) gondoate biosynthesis (anaerobic), (C) adenosine ribonucleotides de novo biosynthesis, and (D) UMP biosynthesis I.

**Supplementary Table S1.** Pairwise comparison of beta diversity from baseline and 18 months samples by PERMANOVA

| **Metric** | **Group** | **Sample size** | ***q*-value** |
| --- | --- | --- | --- |
| Unweighted Unifrac | -20 ℃ | 20 | 0.013 |
|  | -70 ℃ | 20 | 0.584 |
|  | 4 ℃ | 20 | 0.02 |
|  | DNA/RNA Shield + RT | 18 | 0.268 |
|  | OMNIgene + RT | 18 | 0.449 |
|  | RT (20-25 ℃) | 20 | 0.001 |
| Weighted Unifrac | -20 ℃ | 20 | 0.001 |
|  | -70 ℃ | 20 | 0.011 |
|  | 4 ℃ | 20 | 0.001 |
|  | DNA/RNA Shield + RT | 18 | 0.848 |
|  | OMNIgene + RT | 18 | 0.012 |
|  | RT (20-25 ℃) | 20 | 0.001 |

RT, Room temperature
